# Data‐Driven, Patient‐Centred HIV Care Supports Sustained Viral Suppression: A Retrospective Programme Analysis From the Medical Research Foundation of Trinidad and Tobago

**DOI:** 10.1002/jia2.70166

**Published:** 2026-07-25

**Authors:** Nyla P. Lyons, Gregory G. Boyce, K. M. Harvey, Wendy Samaroo‐Francis, Brendon M. Bhagwandeen, Robert J. Edwards

**Affiliations:** ^1^ The Medical Research Foundation of Trinidad and Tobago Port of Spain Trinidad; ^2^ The AIDS Healthcare Foundation Jamaica West Indies; ^3^ University of the West Indies St Augustine West Indies

**Keywords:** data‐driven decision‐making, differentiated service delivery (DSD), HIV care, MRF Centre of Excellence, Trinidad and Tobago, retention in care, viral suppression

## Abstract

**Introduction:**

Achieving and sustaining viral suppression among people living with HIV remains central to global HIV control efforts. Despite global 95–95–95 targets and the scale‐up of differentiated service delivery (DSD) models, many programmes continue to face structural and system‐level barriers to sustained treatment outcomes. Evidence on longitudinal HIV treatment outcomes using routinely collected programme data remains limited in Caribbean settings. This study aimed to describe longitudinal trends in HIV treatment outcomes at the Medical Research Foundation (MRF), a large HIV treatment site in Trinidad and Tobago.

**Methods:**

A retrospective descriptive analysis was conducted using de‐identified electronic medical record (EMR) data for all clients receiving antiretroviral therapy (ART) at MRF. Data were analysed across seven fiscal quarters (FY2024 Q2–FY2025 Q4). The programme delivers differentiated, patient‐centred HIV care supported by routine EMR monitoring and quarterly programme performance reviews. Key indicators included ART coverage, viral load (VL) testing coverage, viral suppression (VL <1000 copies/mL) and treatment interruptions.

**Results:**

The ART cohort ranged from 4913 to 5202 clients over the study period. VL testing coverage remained consistently high, ranging from 95% to 98% per quarter. Viral suppression was sustained at 94%–95%, reaching 95% in three quarters (FY2024 Q3, FY2025 Q3 and FY2025 Q4). Treatment interruptions declined by approximately 50% over time, while the number of clients active on ART remained stable with a gradual increase in later quarters.

**Conclusions:**

This study demonstrates sustained high viral suppression and VL testing coverage within a large HIV treatment programme delivering DSD in Trinidad and Tobago. The reduction in treatment interruptions alongside stable high viral suppression highlights the value of routine programme data for monitoring HIV service delivery in real‐world settings. These findings provide implementation‐relevant insights for strengthening differentiated HIV care models and contribute to the limited evidence base on longitudinal treatment outcomes in Caribbean programme settings.

## 1 Introduction

Despite global 95–95–95 targets, many HIV programmes continue to face structural and system‐level challenges that undermine treatment access and sustained viral suppression, including persistent stigma, socioeconomic vulnerability and conventional service delivery models that remain insufficiently responsive to the long‐term realities of HIV care [1, 2]. Recognizing these constraints, HIV programmes increasingly adopted differentiated service delivery (DSD) models to better align care delivery with patient stability, clinical needs and health system capacity. DSD is a client‐centred approach that simplifies and adapts HIV services across four core domains—who provides care, where services are delivered, when services are provided and what package of services is offered—to improve efficiency while maintaining treatment quality and viral suppression outcomes [3, 4, 5].

As DSD models expand globally, implementation science has emphasized the importance of using routinely collected clinical data to evaluate programme performance and monitor long‐term treatment outcomes in real‐world settings. In the Caribbean region, where many HIV programmes are delivered within resource‐constrained health systems and rely heavily on routine programme reporting and surveillance systems, there is limited evidence on longitudinal treatment outcomes derived from routine clinical data. This reflects a broader implementation science gap in understanding how DSD models perform over time within real‐world programme contexts.

While progress in antiretroviral therapy (ART) coverage and viral suppression across the Caribbean [6] has been made, most available evidence remains descriptive or cross‐sectional, with limited longitudinal evaluation of treatment outcomes within DSD settings. As a result, important gaps remain in understanding the sustained effectiveness of routine care systems in maintaining viral suppression over time within DSD‐informed service delivery structures.

In Trinidad and Tobago, the Medical Research Foundation (MRF) functions as one of the country's largest HIV treatment providers and a national referral centre. Prior to the progressive introduction of DSD models between 2017 and 2025, programme viral suppression was approximately 87% [7]. In response to evolving client needs and persistent structural barriers to care, the programme incrementally expanded decentralized and patient‐centred service delivery approaches aimed at improving long‐term treatment continuity and outcomes. This study, therefore, aimed to describe longitudinal trends in HIV treatment outcomes at the MRF, a large HIV treatment site in Trinidad and Tobago, using routinely collected programme data.

## 2 Methods

### 2.1 Study Design

This was a retrospective descriptive analysis of routinely collected programme data from FY2024 Q2 to FY2025 Q4. Data were extracted from existing clinical and monitoring systems following routine reporting cycles and were not collected prospectively for the purpose of this study.

### 2.2 Study Setting and Service Delivery Model

The MRF is one of the largest HIV treatment providers in the country and functions as a national referral centre in collaboration with the Ministry of Health. HIV care is delivered through a structured DSD model that stratifies clients into stable and non‐stable care pathways based on routine clinical indicators, including viral suppression status, adherence history and clinical stability.

Stable clients are managed through a streamlined care package that includes multi‐month dispensing of ART, extended clinic appointment intervals and fast‐track pharmacy refill systems. Decentralized satellite clinic services provide ART dispensing closer to clients’ communities, with the aim of improving access and reducing stigma associated with facility‐based HIV care. Non‐stable clients, including those with unsuppressed viral load (VL), recent ART initiation or clinical complications, are managed through an intensified follow‐up pathway with more frequent clinical review, enhanced monitoring and multidisciplinary support. ART pick‐up distribution services in South Trinidad are also used to support medication access for selected clients requiring ongoing follow‐up. Between 2017 and 2025, the DSD model was incrementally expanded to include targeted service approaches for men, women, adolescents and youth, and people in prisons or other closed settings.

### 2.3 Data Source

Routine monitoring is conducted using an electronic medical record (EMR) system that captures standard HIV programme indicators. Data are reviewed through structured quarterly programme performance meetings to support quality assurance, service delivery optimization and ongoing programme evaluation.

### 2.4 Data Collection and Validation

Data were extracted from the Medical Research Foundation of Trinidad & Tobago (MRFTT) EMR using standardized programme monitoring queries aligned with national reporting requirements and PEPFAR indicators. De‐identified aggregate datasets were extracted for seven fiscal quarters (Q2 2024–Q4 2025). All extracted datasets underwent routine validation procedures, including duplicate removal, verification of ART status, and consistency checks prior to aggregation and analysis. A multidisciplinary team comprising data coordination, monitoring and evaluation (M&E), statistical staff, and EMR personnel from MRFTT and the Ministry of Health conducted quarterly data quality assessments. This process identified and resolved discrepancies in ART initiation, treatment interruptions, VL testing, missed appointments and patient transfers. Findings were reviewed jointly with clinical, laboratory and pharmacy teams to ensure data accuracy. Validated data were subsequently submitted to the Ministry of Health for routine PEPFAR reporting.

### 2.5 Operational Definitions

#### 2.5.1 Active on ART

Number of clients currently receiving ART and considered active in care at the end of each reporting quarter. This reflects routine programme classification after accounting for deaths, transfers and loss to follow‐up.

#### 2.5.2 VL Testing Coverage

Proportion of clients on ART for ≥3 months with a documented VL result during the reporting period.

#### 2.5.3 Viral Suppression

Defined as a VL <1000 copies/mL among clients receiving ART. The denominator comprised ART clients with a VL result documented in the medical record and/or laboratory information system during the reporting period, while the numerator comprised ART clients with a suppressed VL result (<1000 copies/mL).

#### 2.5.4 Treatment Interruption

Any documented discontinuation of ART during the reporting period, defined as ≥28 days late for a scheduled appointment or medication refill, excluding documented transfers.

#### 2.5.5 Retention on Treatment

Continuous engagement in care, defined as being classified as active on ART across consecutive reporting quarters.

### 2.6 Data Analysis

Data were analysed descriptively using quarterly aggregate counts and proportions. No inferential statistical analyses were conducted. Trends were examined longitudinally across seven fiscal quarters to describe programmatic performance over time.

### 2.7 Ethical Approval

This study involved a retrospective analysis of routinely collected, de‐identified aggregate programme data extracted from the MRFTT EMR system. No direct participant contact was undertaken, and no personally identifiable information were accessed for the purpose of this analysis. Given that the dataset comprised secondary, non‐identifiable programme monitoring data generated through routine HIV service delivery, individual informed consent was not obtained. Ethical approval for the use of secondary programme data for analysis of viral suppression outcomes was previously granted as an exemption by the Campus Research Ethics Committee, University of the West Indies, St Augustine, Trinidad (Ref: CREC‐SA.0910/04/2021).

## 3 Results

The results of our analysis showed that ART coverage remained stable and slightly increased over the seven fiscal quarters (FY2024 Q2–FY2025 Q4). The number of clients on ART ranged from 4913 to 5202 (Table 1). VL testing was consistently implemented across all quarters, with 4703−5089 clients completing VL testing each period, corresponding to coverage of approximately 95%−98%.

**TABLE 1:** Longitudinal HIV treatment indicators by fiscal quarter.

Fiscal quarter	Clients active on ART	VL testing *n* (%)	Viral suppression *n* (%)	Returned to treatment (RTT), *n*
FY2024 Q2	5090	4890 (96%)	4600 (94%)	288
FY2024 Q3	4910	4740 (97%)	4500 (95%)	257
FY2024 Q4	4930	4700 (95%)	4430 (94%)	405
FY2025 Q1	4920	4710 (96%)	4440 (94%)	392
FY2025 Q2	5030	4900 (97%)	4640 (94%)	403
FY2025 Q3	5150	5000 (97%)	4730 (95%)	334
FY2025 Q4	5200	5090 (98%)	4810 (95%)	251

Abbreviations: ART, antiretroviral therapy; FY, fiscal year; VL, viral load; Q, quarter.

**FIGURE 1:**
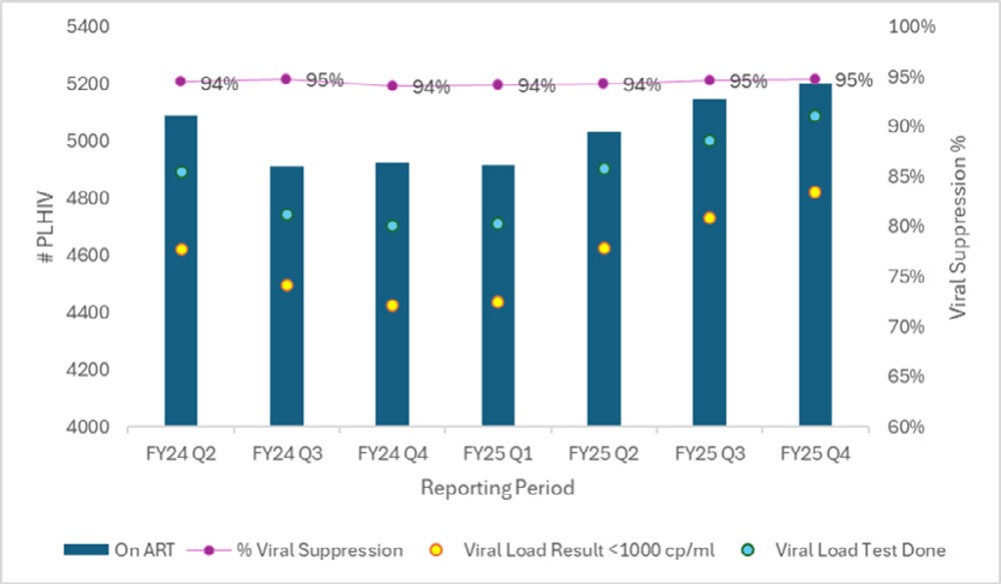
ART coverage, viral load testing and viral suppression among PLHIV at MRFTT across seven fiscal quarters (FY2024 Q2–FY2025 Q4). Abbreviations: ART, antiretroviral therapy; MRFTT, Medical Research Foundation of Trinidad and Tobago; PLHIV, people living with HIV. *Note*: Viral load testing (%) represents the proportion of eligible clients receiving a viral load test during the reporting period. Viral suppression (%) represents the proportion of clients tested with a viral load <1000 copies/mL.

The proportion of clients achieving viral suppression—defined as a VL of <1000 copies/mL—remained consistently high, ranging from 94% to 95%, with the highest suppression rates (95%) reported in FY2024 Q3, FY2025 Q3 and FY2025 Q4 (Figure 1). Treatment interruptions decreased steadily over time, from 446–458 in earlier quarters to 246 in FY2025 Q4, representing an approximate 50% reduction. By June 2025, 5146 clients were active on ART, with approximately 97% completing VL testing in the preceding 12 months and 95% achieving viral suppression. By September 2025, the ART cohort increased to 5202 clients, with VL testing coverage at 98%, viral suppression maintained at 95% and treatment interruptions further reduced (Figure 1).

Fluctuations in the number of clients on ART across FY2024 Q2 to FY2025 Q1 likely reflect routine programme reporting variability, including client transfers and re‐engagement in care, rather than confirmed changes in treatment status. Overall, these findings demonstrate that MRF maintained high VL testing coverage and consistently high viral suppression across seven fiscal quarters. Data on losses to follow‐up, transfers and deaths were not available in the aggregate dataset; therefore, trends should be interpreted with this limitation in mind.

## 4 Discussion

This study demonstrates consistently high ART coverage, VL testing and viral suppression across seven fiscal quarters in a large HIV treatment programme in Trinidad and Tobago. Viral suppression remained stable at 94%–95%, comparable to global benchmarks and countries approaching the UNAIDS 95–95–95 targets. These findings are consistent with a prior cross‐sectional analysis of MRFTT programme data conducted in 2021, which reported viral suppression levels of approximately 94%–95% among clients receiving ART using routine EMR data [8]. Similar outcomes have also been reported in DSD models, which have been associated with sustained viral suppression across diverse settings [9, 10]. The observed stability in outcomes aligns with evidence that routine programme data use and continuous quality improvement approaches can support sustained performance in HIV care systems, although causal inference cannot be established from aggregate programme data.

The results showing reduction in treatment interruptions are consistent with evidence that DSD approaches reduce structural barriers to care through decentralized ART delivery, multi‐month dispensing and simplified service models, which improve continuity of treatment [9, 10]. In addition, peer‐supported and community‐linked models have been shown to improve engagement in care when integrated within routine monitoring systems. Overall, these findings are consistent with global evidence that high viral suppression can be sustained when DSD is combined with routine programme monitoring. However, the observational design and use of aggregate programme data limit causal inference and prevent attribution of outcomes to specific programme components. The findings also align with recent UNAIDS programme documentation describing sustained viral suppression outcomes in Trinidad and Tobago and recognizing the MRF as a model of excellence in HIV care and service delivery within the Caribbean context [11].

A key strength of this analysis is the use of routinely collected programme data across multiple fiscal quarters, allowing the description of longitudinal trends in ART programme performance. However, this descriptive analysis was based on aggregate programme data and was not designed to assess the effects of specific interventions. Consequently, observed trends in VL testing, viral suppression and returned‐to‐treatment outcomes cannot be directly attributed to individual programme components. Aggregate reporting did not include information on new enrolments, deaths, transfers or losses to follow‐up; therefore, changes in the number of clients active on ART should be interpreted cautiously. This analysis was based on routinely collected programme data; findings may be influenced by data completeness and reporting limitations. In addition, the aggregate nature of the data precluded adjustment for potential confounding factors and limited assessment of individual patient trajectories over time.

## 5 Conclusions

This analysis describes longitudinal HIV treatment trends within routine service delivery at a large HIV treatment site in Trinidad and Tobago. High and stable viral suppression, consistent VL testing coverage and sustained programme performance were observed across seven fiscal quarters, alongside a reduction in treatment interruptions.

These findings underscore the value of routinely collected clinical data for monitoring HIV programme performance in real‐world settings and provide implementation‐relevant insights for DSD models. In addition, the results contribute to the limited evidence base from Caribbean settings on longitudinal HIV treatment outcomes within routine programme data systems.

## Author Contributions

NPL conceptualized the study, conducted the data analysis and drafted the initial manuscript. GGB, KMH and RJE provided technical guidance, methodological input and critical review of the manuscript. WS‐F contributed to the technical content and implementation science. All authors contributed to refining the manuscript and approved the final version for submission.

## Conflicts of Interest

The authors declare no conflicts of interest.

## Data Availability

The data that support the findings of this study are available from the corresponding author upon reasonable request.
